# Zinc deficiency impairs axonal regeneration and functional recovery after spinal cord injury by modulating macrophage polarization via NF-κB pathway

**DOI:** 10.3389/fimmu.2023.1290100

**Published:** 2023-11-08

**Authors:** Ken Kijima, Gentaro Ono, Kazu Kobayakawa, Hirokazu Saiwai, Masamitsu Hara, Shingo Yoshizaki, Kazuya Yokota, Takeyuki Saito, Tetsuya Tamaru, Hirotaka Iura, Yohei Haruta, Kazuki Kitade, Takeshi Utsunomiya, Daijiro Konno, V. Reggie Edgerton, Charles Y. Liu, Hiroaki Sakai, Takeshi Maeda, Kenichi Kawaguchi, Yoshihiro Matsumoto, Seiji Okada, Yasuharu Nakashima

**Affiliations:** ^1^ Department of Orthopedic Surgery, Graduate School of Medical Sciences, Kyushu University, Fukuoka, Japan; ^2^ Neurorestoration Center, University of Southern California, Los Angeles, CA, United States; ^3^ Department of Neurological Surgery, Keck School of Medicine, University of Southern California, Los Angeles, CA, United States; ^4^ Department of Energy and Materials, Faculty of Science and Engineering, Kindai University, Osaka, Japan; ^5^ Rancho Research Institute, Los Amigos National Rehabilitation Center, Downey, CA, United States; ^6^ Institut Guttmann. Hospital de Neurorehabilitació, Institut Universitari adscrit a la Universitat Autònoma de Barcelona, Barcelona, Badalona, Spain; ^7^ Department of Orthopaedic Surgery, Spinal Injuries Center, Iizuka, Japan; ^8^ Department of Orthopaedic Surgery, Fukushima Medical University, Fukushima, Japan; ^9^ Department of Orthopedic Surgery, Osaka University Graduate School of Medicine, Osaka, Japan

**Keywords:** spinal cord injury, zinc deficiency, zinc supplementation, NF-κB, pro-inflammatory like macrophage, axonal regeneration

## Abstract

**Background:**

Spinal cord injury (SCI) is a devastating disease that results in permanent paralysis. Currently, there is no effective treatment for SCI, and it is important to identify factors that can provide therapeutic intervention during the course of the disease. Zinc, an essential trace element, has attracted attention as a regulator of inflammatory responses. In this study, we investigated the effect of zinc status on the SCI pathology and whether or not zinc could be a potential therapeutic target.

**Methods:**

We created experimental mouse models with three different serum zinc concentration by changing the zinc content of the diet. After inducing contusion injury to the spinal cord of three mouse models, we assessed inflammation, apoptosis, demyelination, axonal regeneration, and the number of nuclear translocations of NF-κB in macrophages by using qPCR and immunostaining. In addition, macrophages in the injured spinal cord of these mouse models were isolated by flow cytometry, and their intracellular zinc concentration level and gene expression were examined. Functional recovery was assessed using the open field motor score, a foot print analysis, and a grid walk test. Statistical analysis was performed using Wilcoxon rank-sum test and ANOVA with the Tukey-Kramer test.

**Results:**

In macrophages after SCI, zinc deficiency promoted nuclear translocation of NF-κB, polarization to pro-inflammatory like phenotype and expression of pro-inflammatory cytokines. The inflammatory response exacerbated by zinc deficiency led to worsening motor function by inducing more apoptosis of oligodendrocytes and demyelination and inhibiting axonal regeneration in the lesion site compared to the normal zinc condition. Furthermore, zinc supplementation after SCI attenuated these zinc-deficiency-induced series of responses and improved motor function.

**Conclusion:**

We demonstrated that zinc affected axonal regeneration and motor functional recovery after SCI by negatively regulating NF-κB activity and the subsequent inflammatory response in macrophages. Our findings suggest that zinc supplementation after SCI may be a novel therapeutic strategy for SCI.

## Introduction

Traumatic spinal cord injury (SCI) is a devastating disease that causes severe motor and sensory dysfunction, significantly reducing quality of life (1). Mechanical trauma rapidly causes disruption of the blood-brain barrier, neuronal death, axonal damage and demyelination, followed by a cascade of secondary injuries that expand the inflammatory response, which is characterized by infiltration of circulating cells such as macrophages and neutrophils at the epicenter of the injury (2, 3). Due to the limited endogenous regenerative and reparative capacity of the central nervous system (CNS), it is important to identify SCI exacerbating factors that can be intervened upon (4, 5). Age, blood pressure, and infection are each considered as prognostic factors for SCI, but factors that enable therapeutic intervention remain to be fully elucidated (5–8).

Zinc, an essential trace element, has been widely reported to play a role in regulating inflammation in recent years (9–11). For example, zinc deficiency exacerbates inflammation such as diarrhea and increases mortality from inflammatory diseases such as sepsis (9, 11–14), while zinc supplementation has been reported to improve inflammation and decrease the duration and severity of inflammatory diseases such as respiratory tract infections and sepsis (15–18).

Macrophages, immune cells that infiltrate into CNS, form a line of defense after exposure to invading pathogens and tissue damage (19). After SCI, activated macrophages express cytokines such as tumor necrosis factor-a (TNF-a), interleukin-6 (IL-6) and IL-1b and promote subsequent inflammatory responses (20, 21). Recently, we have shown that macrophage activation is associated with neuropathological outcomes in SCI (22). Although the exact mechanism of macrophage activation is not yet fully understood, several basic studies have reported that zinc is involved in macrophage activation (23). For example, it has been reported that the expression of inflammatory cytokines in macrophage is increased in zinc-deficient rodents, which worsens the prognosis of sepsis (12). In addition, it has been reported that zinc significantly improves the macrophage phagocytic capacity (23, 24). Considering that many elderly people and most chronic disease patients are zinc deficient, zinc may represent a novel therapeutic target to alter macrophage responses and regulate inflammation after SCI (25, 26).

In this study, we investigated the effects of zinc on the pathophysiology and motor function after SCI using an experimental mouse model and *in vitro* experiments. Using physiological and histological analysis and cell type-specific gene expression analysis by flow cytometry, we found that the low zinc status promoted nuclear translocation of NF-κB in macrophages, which altered macrophage phenotype, enhanced inflammatory cytokine expression, inhibited axonal regeneration and worsened motor functional outcome after SCI. Moreover, we showed that zinc supplementation to zinc-deficient mice improved inflammation, axonal regeneration and motor functional recovery after SCI, indicating direct relationship between zinc deficiency and worse outcome. These results suggest that zinc supplementation is an effective treatment for SCI.

## Materials and methods

### Mice

Adult female C57BL/6 wild-type mice aged 8-10 weeks were used. Mice were kept under constant conditions of a 12-hour light/dark cycle and a room temperature of 23°C ± 2°C, with *ad libitum* access to food and water (5). To create a mouse model of zinc deficiency, mice were fed a zinc-deficient diet (Kyudo company, Saga, Japan). For zinc supplementation, water containing a high volume of zinc (Nacalai Tesque, Kyoto, Japan) was prepared and provided. Mice were excluded from this study if they died, developed infections resistant to antibiotic treatment, or developed significant autophagy. All animal experiments were approved by our university’s Animal Experimentation Ethics Committee and were conducted in compliance with the National Institutes of Health guidelines for the Care and Use of Animals. All efforts were taken to reduce the number of animals used and to minimize animal suffering.

### Spinal cord injury

Mice were anesthetized by intraperitoneal injection of mixed anesthesia with midazolam (4 mg/kg), butorphanol tartrate (5 mg/kg), and medetomidine hydrochloride (0-3 mg/kg). After laminectomy at the 10th thoracic level, we exposed the dorsal surface of the dura mater and induced a contusion injury using the Infinite Horizons Impactor (Precision Systems Instrumentation, Lexington, KY, USA) (27). Injury with Impactor was performed at force setting of 70kdyn, its accepted displacement range was 580-620 microns (3). After SCI, the surrounding muscles were sutured, the skin was closed with suture wound clips, and the mice were placed in a temperature-controlled chamber during recovery from anesthesia until thermoregulation was re-established (1). Motor function was assessed using a locomotor open-field rating scale, BMS (4). Footprint analysis was performed as previously reported (6). We dipped the forelimbs and hindlimbs of mice in red and green dyes, respectively. For the grip-walk test, we evaluated each mouse using a 50-cm grid with three patterns: easy (50 steps, 1 cm apart), medium (removed every third step), and hard (removed every other step) (5). The total number of grips for the three patterns was used for analysis (28). To collect cell-free serum, 0.6 ml of blood was collected by cardiac puncture. After standing upright for 30 minutes at room temperature and 6 hours at 4°C, samples were centrifuged at 4000 rpm for 15 minutes at 4°C. The supernatant was quickly removed and immediately stored at -30°C until further testing (3). Serum zinc concentrations were measured using the Metallo assay Zn LS Kit (ZN02M, Metallogenics, Chiba, Japan) according to the manufacturer’s protocol.

### Histopathological examination

Mice were reanesthetized and fixed transcardially with 4% paraformaldehyde. The spinal cord was then removed, dehydrated, and embedded in an optimal cutting temperature (OCT) compound. Frozen sections were cut at 16 μm in the sagittal plane (1). Primary antibodies were applied to the sections at 4°C, followed by incubation of the sections with FluoZin3 (F24195, 10 μM; Invitrogen, Carlsbad, CA, USA) and Alexa Fluor-conjugated secondary antibody (1:200; Invitrogen) and Hoechst 33258. THP-1 cells were also fixed and dehydrated, then the antibodies were applied similarly. All images were captured with a BZ-9000 digital microscope system (Keyence, Osaka, Japan) or fluorescence microscope equipped with a digital camera (BX51, Olympus, Tokyo, Japan). For quantitative evaluation, sagittal sections were selected from positions 0.08 mm, 0.16 mm, 0.24 mm, and 0.32 mm to the left and right of the midline and used for analysis. To compare the LFB positive area, sagittal sections of injured spinal cord were selected and measured with the software of BZII-Analyzer (Keyence) to calculate the ratio of LFB positive area to normal sections, as previously described (5, 29, 30). Cell counts and the GAP43-positive area and specific color area were determined using the National Institutes of Health ImageJ software program (National Institutes of Health, Bethesda, MD, USA).

### Flow cytometry

Spinal cord samples (6.0 mm long, centered on the lesion) were prepared for flow cytometry as previously described (3, 29). These samples were stained with anti-CD45 (103,131, Biolegend, San Diego, CA, USA), anti-Gr-1 (108,415, Biolegend) and anti-CD11b (101,211, Biolegend). Cells were then counterstained with FluoZin-3 AM (Invitrogen). Intracellular zinc levels were compared based on mean fluorescence intensity (MFI). Samples were analyzed with a FACSAria II flowcytometer (BD Biosciences), San Jose, CA, USA) and analyzed with the FACSDiva software program (BD Biosciences) (3). THP-1 cells were stained with anti-CD68 (Bio-Rad, Hercules, CA, USA) and then counterstained with Alexa Fluor-conjugated secondary antibody (1:200; Invitrogen) and FluoZin-3 AM (Invitrogen) and analysis was performed with Attune NxT Flow Cytometer (Thermo Fisher Scientific, Waltham, MA, USA).

### Quantitative reverse transcription-PCR

Total RNA was isolated from spinal cord tissue using the RNeasy Mini Kit (74,004, Qiagen, Hilden, Germany) and from FACS-sorted macrophages and THP-1 cells 4 h after LPS treatment using the RNeasy Micro Kit (74,104, Qiagen) (3). In order to synthesize complementary DNA (cDNA), we performed reverse transcriptase reactions using the PrimeScript first-strand cDNA Synthesis Kit (6110A, Takara Bio, Otsu, Japan). Quantitative real-time PCR (qRT-PCR) was performed by using primers specific for the target gene (see Additional file 1) and SYBR Premix Dimmer-Eraser (RR091A, Takara Bio, Shiga, Japan). Data were normalized against glyceraldehyde-3-phosphate dehydrogenase (GAPDH) levels.

### THP-1 cell culture

The human monocytic cell line THP-1 (Japanese Collection of Research Bioresource, Osaka, Japan) was cultured in RPMI medium 1640 containing 10% fetal bovine serum, 1% penicillin-streptomycin and 2 mM L-glutamine (3). In order to differentiate THP-1 cells into macrophages, THP-1 cells were incubated with phorbol 12-myristate 13-acetate (PMA) (27547-14, Nacalai Tesque) at a concentration of 10 ng/mL for 72 hours (12). The concentration of zinc in the culture medium was adjusted by adding N,N,N′,N′-tetrakis (2-pyridinylmethyl)-1,2-ethanediamine (TPEN) (P4413, Sigma-Aldrich, St. Louis, MO, USA) or zinc (Nacalai Tesque). THP-1 macrophages in each culture were treated with LPS at a concentration of 1 μg/ml for 1 hour, changed to the respective culture medium containing LPS and incubated for 3 hours before analysis. Flow cytometry, mRNA extraction, zinc concentration measurements, or immunocytochemical staining were performed as previously reported (12). For cyto-immunofluorescence staining, THP-1 macrophages were incubated with 10 μM FluoZin-3 AM with Pluronic F-127 (P3000MP, Invitrogen) for 60 min at 37°C and then analyzed by confocal microscopy (12).

### Statistics

Statistical evaluation between the two groups was performed with Wilcoxon’s rank sum test. ANOVA with the Tukey-Kramer *post hoc* test was used for multiple comparisons between groups. For all statistical analyses, the significance level was set at 0.05. Values for each group were presented as mean ± standard error of the mean (SEM). All statistical analyses were performed using the JMP software program (version 15; SAS Institute, SAS Institute, Cary, NC, USA).

## Results

### Zinc deficiency promotes nuclear translocation of NF-κB in macrophages with change of macrophage phenotype and exacerbates subsequent inflammatory response

**Figure 1:**
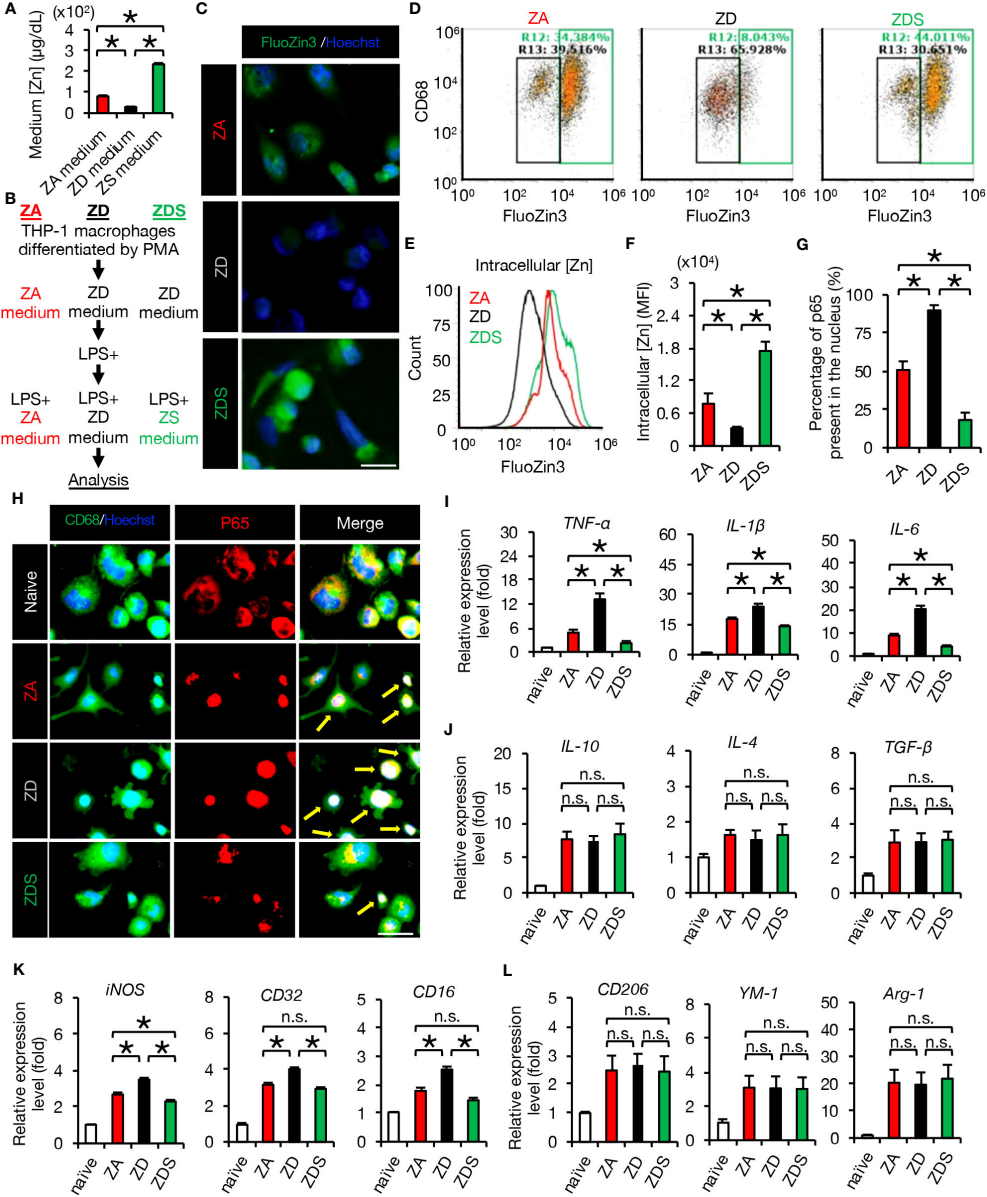
Zinc negatively regulates NF-κB activity and inflammatory responses. **(A)** Zinc concentration in culture medium (n = 4 per group). **(B)** Protocol for analysis of THP-1 macrophages *in vitro*. **(C)** Image analysis of macrophages using the cell-permeable zinc indicator FluoZin-3 (green). The nuclei are counterstained with Hoechst 33258 dye (blue). **(D, E)** Flowcytometric analysis and histogram analysis of FluoZin-3-positive macrophages. **(F)** Flowcytometric analysis. The relative levels of intracellular zinc were compared based on the mean fluorescence intensity (MFI) (n = 6 per group). Significant differences in zinc influx into the cells were observed after 4 hours of LPS stimulation. **(G)** To find out how much of all NF-κB p65 is in the nucleus, we measured the area of all p65 (1H, red area in the p65 image) and the area of p65 that has translocated into the nucleus (1H, white area in the Merge image) using the ImageJ function to measure the area of specific colors. The results showed significant differences among the three groups. **(H)** Representative images of immunocytochemical analysis of NF-κB p65 translocation into the nucleus of macrophages after LPS stimulation, stained with CD68 (green), NF-κB p65 (red) and Hoechst (blue). Arrows indicate nuclear translocation of p65 (white). **(I)** Gene expression of pro-inflammatory cytokine of macrophages cultured in each zinc condition. (n = 6 per group). **(J)** Gene expression of anti-inflammatory cytokines of macrophages cultured in each zinc condition. (n = 6 per group). **(K)** Gene expression of pro-inflammatory like macrophage markers (n = 6 per group). **(L)** Gene expression of anti-inflammatory like macrophage markers (n = 6 per group). Images shown in **(C, H)** are representative of 6 sections. Scale bar = 20μm **(C)** and 30μm **(H)**. In qRT-PCR analysis, each group was normalized to GAPDH values. *P < 0.05, ANOVA with the Tukey-Kramer *post hoc* test. n.s., not significant. Error bar indicates mean ± SEM. ZA, zinc-adequate; ZD, zinc-deficient; ZDS, zinc deficient followed by supplementation.

In acute SCI, activated macrophages enhance and propagate the subsequent inflammatory response (21). To evaluate the inflammatory response of macrophages under different zinc conditions, we first prepared mediums with different zinc concentrations in which we incubated THP-1 macrophages as described in Methods. The zinc concentrations of zinc-adequate (ZA), zinc-deficient (ZD) and zinc-supplementation (ZS) medium are 81.7 ± 1.2, 29.4 ± 0.6, and 233 ± 1.5 (μg/dl), respectively (**Figure 1A**). The analysis protocols for macrophages *in vitro* are as follows: for Zinc deficient followed by supplementation (ZDS) analysis, THP-1 macrophages differentiated using PMA were cultured in ZD medium, then LPS was added, and 1 hour later the medium was replaced with ZS medium containing LPS and analyzed 3 hours later. For ZA and ZD analysis, THP-1 macrophages differentiated using PMA were cultured in ZA and ZD medium, respectively, then LPS was added, and 1 hour later the medium was replaced with ZA and ZD medium containing LPS and analyzed 3 hours later as described in Methods (**Figure 1B**).

In macrophages after LPS addition and macrophages after SCI, it is known that zinc is taken up into intracellular space via the zinc importer SLC39A8 (ZIP8) (3). Therefore, we first evaluated intracellular zinc levels in these three groups by immunocytochemical staining and flow cytometry using the cell-permeable zinc indicator FluoZin-3 (12). As a result, the intracellular zinc levels of ZD macrophages were significantly decreased and those of ZDS macrophages were significantly increased compared to the intracellular zinc levels of ZA macrophages (**Figures 1C, E, F**). Furthermore, the percentage of zinc-positive population of ZD macrophages was significantly decreased and those of ZDS macrophages were significantly increased compared to the percentage of zinc-positive population of ZA macrophages (**Figure 1D**). These results indicate that we have successfully developed a protocol for analysis of macrophages in different intracellular zinc status.

Since zinc is reported to negatively regulate the activity of NF-κB p65 (12, 23), we examined whether or not zinc deficiency and zinc supplementation influence the nuclear translocation of NF-κB and the following inflammatory pathology in macrophages. As a result, immunocytochemical staining revealed a significantly increased nuclear translocation of NF-κB in ZD macrophages and a significantly decreased nuclear translocation of NF-κB in ZDS macrophages compared to ZA macrophages, respectively (**Figures 1G, H**). In addition, qRT-PCR revealed that gene expression of pro-inflammatory cytokines such as *TNF-α*, *IL-1β*, and *IL-6* was significantly increased in ZD macrophages and significantly decreased in ZDS macrophages compared to ZA macrophages (**Figure 1I**). In contrast, gene expression of anti-inflammatory cytokines such as *IL-10, IL-4* and *TGF-β* did not differ significantly among these three groups (**Figure 1J**). Moreover, since NF-κB is reported to be involved in the polarization of pro-inflammatory like macrophages (31), we examined whether or not the intracellular zinc level affects the polarization of macrophages in these three groups. As a result, gene expression of the pro-inflammatory like macrophage marker *iNOS* increased significantly in ZD macrophages and decreased significantly in ZDS macrophages compared to ZA macrophages (**Figure 1K**). In addition, gene expression of pro-inflammatory like macrophage markers such as *CD32* and *CD16* was significantly increased in ZD macrophages compared to ZA macrophages, whereas these changes were cancelled in ZDS macrophages (**Figure 1K**). In contrast, gene expression of anti-inflammatory like macrophage markers such as *CD206, YM1* and *Arginase-1* did not differ significantly among these three groups (**Figure 1L**). These results suggest that zinc deficiency promotes nuclear translocation of NF-κB in macrophages, thereby promoting polarization to pro-inflammatory like macrophages and subsequent inflammatory responses, and that zinc supplementation cancels these responses induced by zinc deficiency.

### Zinc regulated NF-κB activity and subsequent inflammatory response after SCI

**Figure 2:**
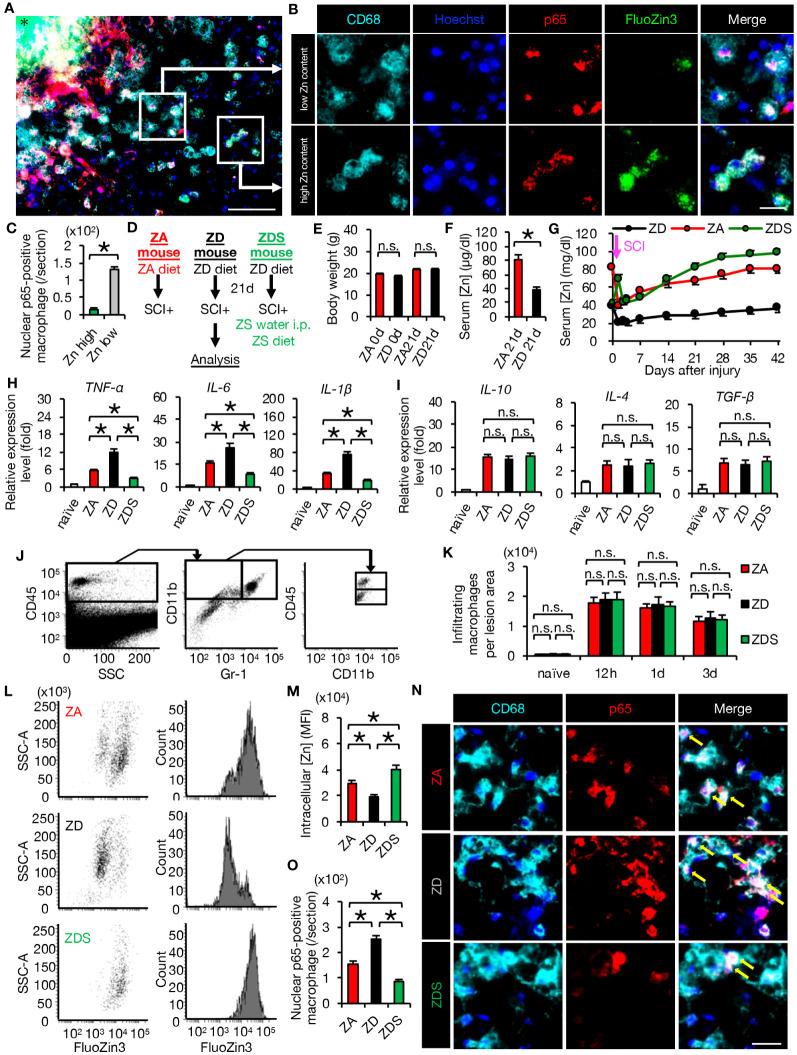
Zinc suppressed nuclear translocation of NF-κB in macrophages after SCI **(A, B)** Representative images of immunocytochemical analysis of the perilesional areas in normal-fed mice at 4 dpi, stained with CD68 (cyan), Hoechst (blue), NF-κB p65 (red) and FluoZin3 (green). Nuclear translocation of NF-κB p65 was observed in macrophages with low intracellular zinc, while it was not observed in macrophages with high intracellular zinc. * indicates the lesion epicenter. **(C)** Quantification of nuclear p65-positive numbers in macrophages with low and high intracellular zinc content. **(D)** Animal experiment protocol. **(E)** There were no differences in body weight by diet type (n = 10 per group). **(F)** Serum zinc concentrations before SCI (n = 6 per group). **(G)** Time course of serum zinc concentrations (n = 6 per group at each time point). **(H)** In the spinal cord at 3 dpi, gene expression of pro-inflammatory cytokine, which was increased by zinc deficiency, was decreased by zinc supplementation. (n = 6 per group). **(I)** Gene expression of anti-inflammatory cytokines was not significantly different among the three groups at 3 dpi (n = 6 per group). **(J)** Flowcytometric analysis. The CD11b^high^/Gr-1^nega-int^/CD45^high^ macrophage fraction in the injured spinal cord (upper box). **(K)** Changes in the number of macrophages in the lesion (n = 6 per group). **(L, M)** Flowcytometric analysis. Intracellular zinc levels in macrophages at 3 dpi (n = 6 per group). **(N, O)** CD68 (cyan) and p65 (red) double-positive macrophages in the perilesional areas. The nuclei are counterstained with Hoechst 33258 dye (blue). Arrows indicate nuclear translocation of p65. The number of nuclear translocation of NF-κB was increased in ZD mice and decreased in ZDS mice compared to ZA mice at 4 dpi. Images shown in **(A, B, N)** are representative of 8 sections per 6 mice. Scale bar = 150μm **(A)**, 50μm **(B)** and 70μm **(N)**. In qRT-PCR analysis, each group was normalized to GAPDH values. *P < 0.05, Wilcoxon’s rank sum test, ANOVA with the Tukey-Kramer *post hoc* test. n.s., not significant. Error bar indicates mean ± SEM.

In order to examine the effect of zinc on inflammation *in vivo*, we first observed the injured spinal cords of mice fed a normal diet using immunostaining at 4 days post-injury (dpi). Interestingly, nuclear translocation of NF-κB was increased in macrophages with low intracellular zinc content compared to those with high intracellular zinc content, indicating that zinc suppresses the nuclear translocation of NF-κB in macrophages after SCI (**Figures 2A–C**).

For further analyses, we created SCI mouse models with different zinc status. ZA or ZD mice were fed the ZA or ZD diets for 3 weeks respectively, followed by SCI. ZA or ZD mice were then fed the ZA or ZD diet, respectively, for up to 6 weeks until each analysis. ZDS mice were fed a ZD diet for 3 weeks followed by SCI, and injected intraperitoneally high-concentration zinc immediately after SCI. ZDS mice were then received the ZA diet and high-concentration zinc water orally for up to 6 weeks until each analysis (**Figure 2D**). As a result, a significant decrease in serum zinc concentration was observed in the ZD and ZDS mice before SCI compared to the ZA mice, however, no significant changes in body weight were observed among these mouse groups (**Figures 2E–G**). The ZDS mouse group also showed a transient increase in serum zinc concentration after intraperitoneal administration of high-concentration zinc water, followed by a gradual increase in serum zinc concentration (**Figure 2G**). These results indicate that we had successfully developed SCI models with different serum zinc concentration.

**Figure 3:**
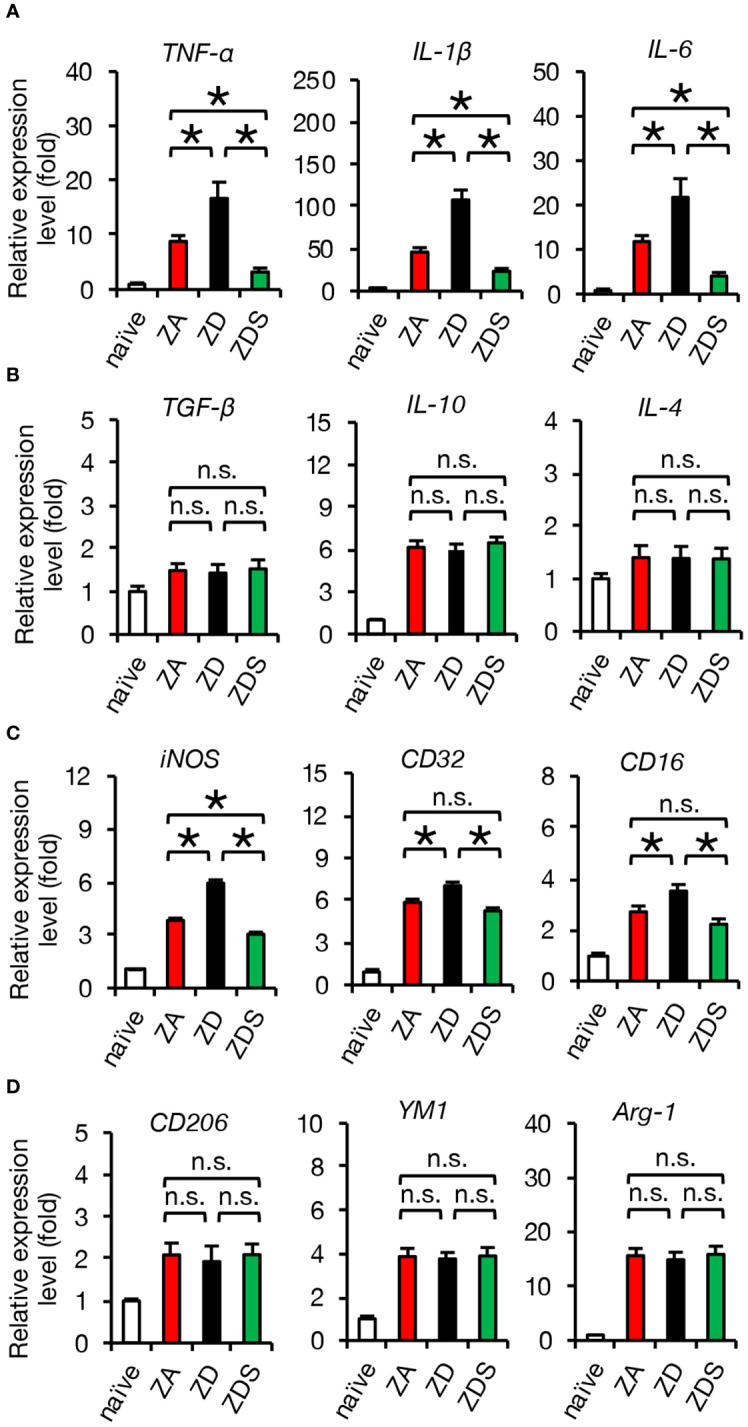
Gene expression of isolated macrophages after SCI is altered by systemic zinc status. **(A)** Gene expression of pro-inflammatory cytokine, which was increased by zinc deficiency, was improved by zinc supplementation (n = 6 per group). **(B)** Gene expression of anti-inflammatory cytokines was not significantly different among the three groups at 3 dpi (n = 6 per group). **(C)** Gene expression of pro-inflammatory like macrophage markers iNOS, which was increased by zinc deficiency, was improved by zinc supplementation (n = 6 per group). **(D)** Gene expression of anti-inflammatory like macrophage markers was not significantly different among the three groups at 3 dpi (n = 6 per group). In qRT-PCR analysis, each group was normalized to GAPDH values. *P < 0.05, ANOVA with the Tukey-Kramer *post hoc* test. n.s., not significant. Error bar indicates mean ± SEM.

Next, we performed gene expression analysis of inflammatory cytokines to assess whether the zinc status altered inflammation after SCI. A qRT-PCR of the injured spinal cord showed that gene expression of pro-inflammatory cytokines significantly increased in ZD mice and significantly decreased in ZDS mice compared to ZA mice (**Figure 2H**). On the other hand, gene expression of the anti-inflammatory cytokines was not significantly different among these three groups (**Figure 2I**). To clarify the regulatory mechanism underlying the zinc altered the expression changes of pro-inflammatory cytokines in injured spinal cord, we selectively isolated 5000 infiltrating macrophages from injured spinal cord using a cell sorter and performed gene expression analysis, as described in our earlier studies (3, 5, 29). In brief, macrophages were selectively isolated as a CD11bhigh/Gr-1nega-int/CD45high population after dead cells were removed using DAPI (**Figure 2J**). Consequently, there was no significant difference in the number of infiltrating macrophages among the three groups (**Figure 2K**), however, there were significant differences among the three groups in the amount of intracellular zinc concentration and the number of nuclear translocations of NF-κB in macrophages (**Figures 2L–O**). In addition, consistent with the *in vitro* results, in the isolated macrophages, gene expression of pro-inflammatory cytokines such as *TNF-α*, *IL-1β* and *IL-6* was significantly different among the three groups (**Figure 3A**), while gene expression of anti-inflammatory cytokines such as *TGF-β, IL-10* and *IL-4* was not significantly different among these three groups (**Figure 3B**). Furthermore, gene expression of the pro-inflammatory like macrophage marker *iNOS* was significantly different among the three groups (**Figure 3C**), while gene expression of anti-inflammatory like macrophage markers such as *CD206, YM1*, and *Arginase-1* was not significantly different among these three groups (**Figure 3D**). These results suggest that zinc suppresses the nuclear translocation of NF-κB after SCI, thereby altering macrophage polarization and consequently reducing inflammatory responses.

### Zinc supplementation significantly improved axonal regeneration and functional recovery after SCI

**Figure 4:**
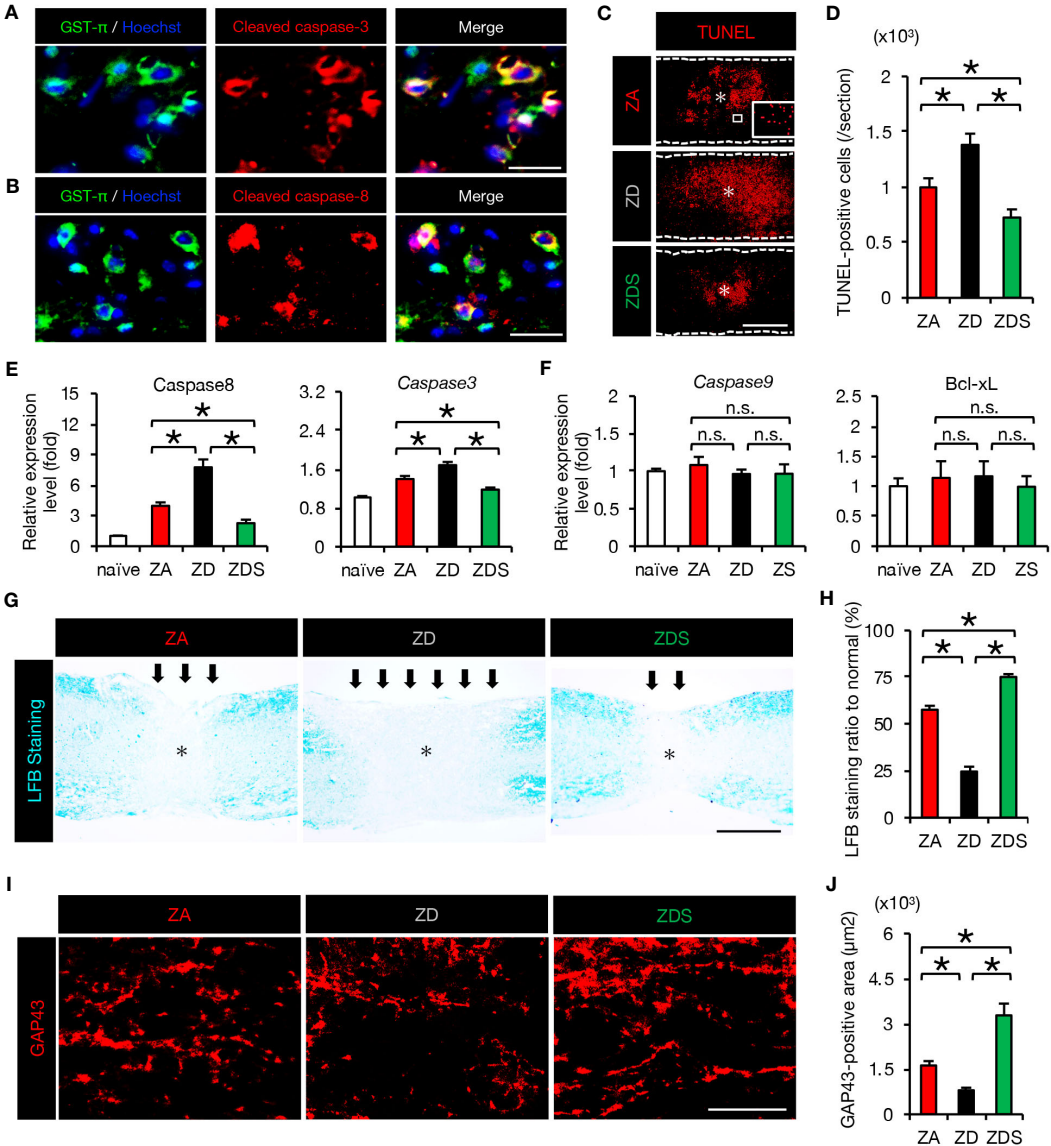
The increase in apoptosis and demyelinated areas caused by zinc deficiency attenuated with zinc supplementation. **(A)** GST-π (green) and cleaved caspase-3 (red) double-positive oligodendrocytes in the perilesional areas. at 4 dpi. The nuclei are counterstained with Hoechst 33258 dye (blue). **(B)** GST-π (green) and cleaved caspase-8 (red) double-positive oligodendrocytes in the perilesional areas. at 4 dpi. The nuclei are counterstained with Hoechst 33258 dye (blue). **(C)** TUNEL staining of the section at 4 dpi. Inset: TUNEL-positive cells. * indicates the lesion epicenter. **(D)** Quantification of the TUNEL-positive apoptotic cells in the lesion at 4 dpi (n = 8 per group). **(E, F)** Expression of apoptosis-related genes at 3 dpi (n = 6 per group). **(G, H)** LFB staining at 42 days after SCI showed a greater demyelinated area (arrows) in ZD mice and a smaller demyelinated area in ZDS mice compared to ZA mice. * indicates the lesion epicenter. **(I)** GAP43 staining in the caudal area of the lesion at 42 dpi. **(J)** Quantification of the GAP43-positive area per 1.0 × 10^5^ µm^2^ (n = 8 per group). The asterisk indicates the epicenter of the lesion. Images shown in **(A, B, G, I)** are representative of 8 sections per 6 mice. Scale bar = 40μm **(A, B)**, 500μm **(C, G)** and 40μm **(I)**. In qRT-PCR analysis, each group was normalized to GAPDH values. *P < 0.05, ANOVA with the Tukey-Kramer *post hoc* test. n.s., not significant. Error bar indicates mean ± SEM.

Since *TNF-α* has been reported as inducing apoptosis of neurons and oligodendrocytes via the caspase-8/caspase-3 pathway (5, 29), we examined the activation of apoptotic cascade in SCI mice fed a normal diet. As a result, we observed the presence of double immunostained cells of glutathione S-transferase p (GST-p), a marker of mature oligodendrocytes, and cleaved caspase 3/caspase 8 (activated caspase 3/caspase 8) around the lesion at 4 dpi (**Figures 4A, B**). Furthermore, TUNEL staining revealed that the number of peri-lesional apoptotic cells was significantly increased in ZD mice and significantly decreased in ZDS mice compared to ZA mice (**Figures 4C, D**). Along with the extrinsic apoptotic pathway mediated by *caspase-8*, another intrinsic apoptotic pathway mediated by *caspase-9* and *Bcl-xL* is known (5). Therefore, the expression of the factors involved in both the extrinsic and the intrinsic apoptosis pathways was assessed. The expression of *caspase-8* and *caspase-3* in the injured spinal cord was significantly increased in ZD mice and significantly decreased in ZDS mice compared to ZA mice, while *caspase-9* and *Bcl-xL* expression was comparable among the three groups (**Figures 4E, F**). This suggests that after SCI, zinc deficiency promotes neuronal apoptosis not through mitochondrial intrinsic pathway but through extrinsic pathway mediated by *TNF-α*. Oligodendrocyte apoptosis after SCI is known to be associated with demyelination of the injured spinal cord and subsequent impaired functional recovery (5, 32). Here, we observed a greater extent of demyelination in ZD mice and a smaller extent of demyelination in ZDS mice compared to ZA mice (**Figures 4G, H**). In addition, it has also been reported that *TNF-α* and macrophage inflammation are associated with inhibition of axonal regeneration and subsequent functional recovery (19, 33). With regard to axonal regeneration, immunostaining with an antibody against GAP43 (1), a marker of regenerating axons, revealed that the number of GAP43-positive axons in the caudal area of the lesion was significantly increased in ZDS mice and significantly decreased in ZD mice, compared to ZA mice (**Figures 4I, J**).

**Figure 5:**
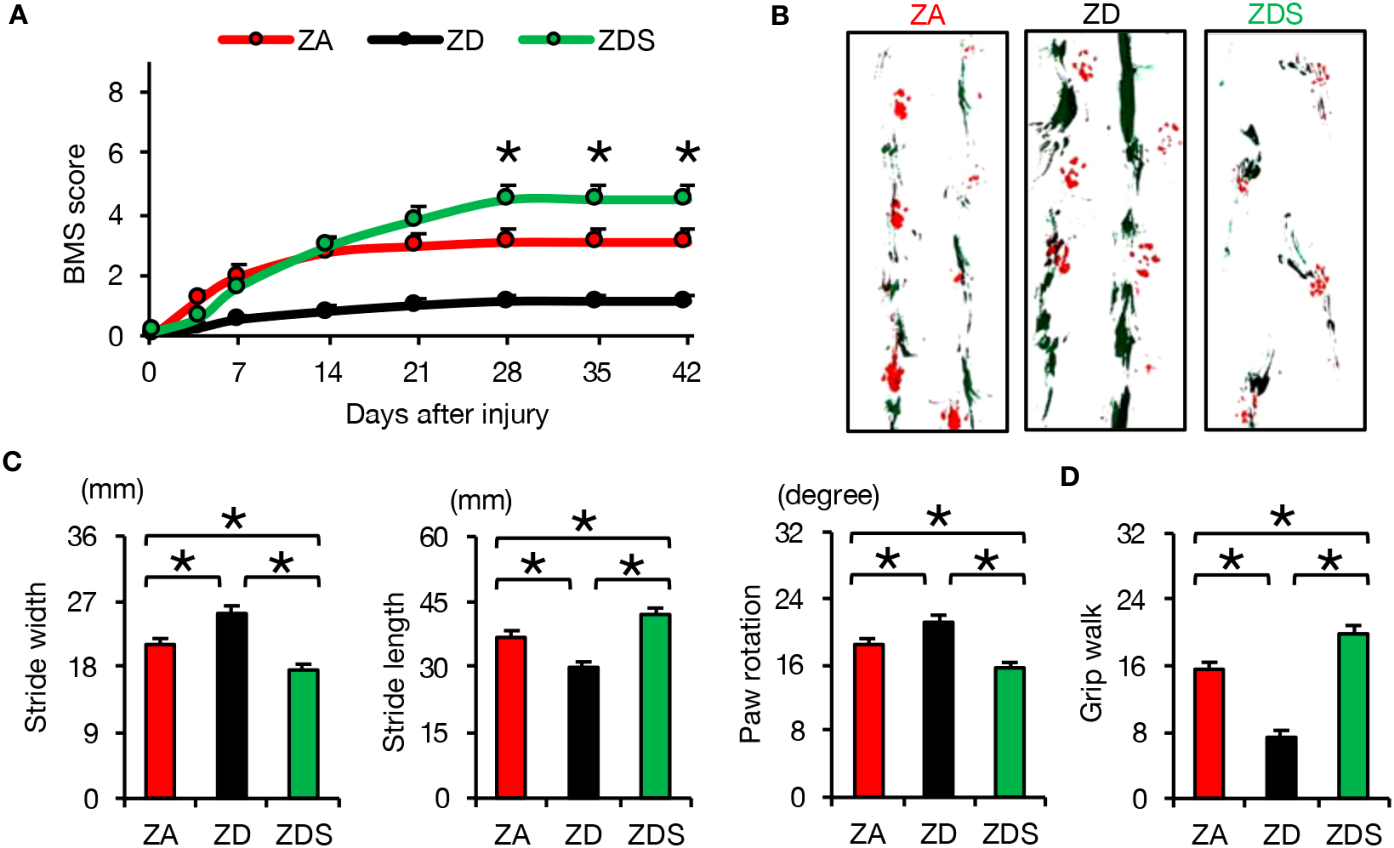
Zinc supplementation after SCI improved motor function worsened by zinc deficiency. **(A)** The time course of functional recovery according to the BMS score after SCI (n =15 per group). **(B, C)** The results of the footprint analyses (red, forepaws; green, hindpaws) at 42 dpi (n =12 per group). **(D)** The results of the grid walk test at 42 dpi (n =12 per group). *P < 0.05, ANOVA with the Tukey-Kramer *post hoc* test. Error bar indicates mean ± SEM.

In order to evaluate the clinical application of zinc for the treatment of SCI, we finally assessed the effect of zinc on the recovery of motor function. ZD mice exhibited poorer functional outcomes and ZDS mice exhibited better functional outcomes compared to ZA mice, as measured by the Basso Mouse Scale (BMS) scores, footprint analysis, and the Grip Walk test after SCI (**Figures 5A–D**). These objective results reinforce the notion that zinc supplementation is a feasible treatment to improve functional recovery after SCI.

## Discussion

In this study, we revealed that zinc deficiency exacerbates the inflammatory response after SCI, thereby inhibiting axonal regeneration and worsening motor function. Conversely, zinc supplementation ameliorated these responses induced by zinc deficiency and improved motor function. Moreover, we investigated the mechanisms underlying the inflammatory regulation by zinc and clarified that zinc inhibits the nuclear translocation of NF-κB, thereby suppressing macrophage polarization to pro-inflammatory like phenotypes and the subsequent expression of pro-inflammatory cytokines. These findings highlight the importance of zinc supplementation to improve motor function after SCI.

The population with zinc deficiency is considered large. According to WHO, in developing countries, 2 billion people are zinc deficient and zinc deficiency is the fifth leading cause of death and disease (11, 18). In developed countries, zinc deficiency in the elderly is considered common, and indeed it has been reported that about 30-40% of the elderly population may be zinc deficient (34). The high prevalence of low zinc concentration in elderly people is well documented (35, 36). Thus, it is estimated that zinc deficiency affects about one-third of the world population (11). In fact, our previous report (Kijima et al., 2019) also confirmed that there are some patients who have low zinc status during the acute phase of SCI (3). Considering the high prevalence of zinc deficiency and chronic disease worldwide and the growing number of elderly patients with SCI, there is potentially a large population of SCI patients who present with low zinc status prior to SCI and require zinc supplementation therapy (3, 35).

In the present study, in inflammatory conditions, low zinc status enhanced the nuclear translocation of NF-κB (**Figures 1H**, **2N**), resulting in macrophage differentiation into pro-inflammatory like phenotypes and increased expression of pro-inflammatory cytokines (**Figure 3**). It was reported that NF-κB binds to the *TNF-α* promoter, indicating that NF-κB directly modulates *TNF-α* transcription in macrophages (5, 37). *TNF-α* has also been reported to be associated with induction of apoptosis of neural cells, including oligodendrocytes and neurons, and inhibition of axonal regeneration after SCI (2, 19, 33). In addition, the previous report by Bao et al. that *TNF-α* with zinc deficiency increases Caspase-8 activity and that zinc administration prevents this zinc-deficiency-induced apoptosis supports our results (38). Therefore, the overexpression of *TNF-α* observed in macrophages due to the activation of NF-κB in the low zinc state is expected to contribute to poor functional outcome after SCI by increasing apoptosis of neural cells and inhibiting axonal regeneration (**Figures 4**, **5**).

Regarding the mechanism by which macrophages differentiate into pro-inflammatory like phenotypes, TLR4 is considered to possibly play a role. After SCI, necrotic cells release damage-associated molecular patterns (DAMPs) such as heat shock proteins, fibronectin, high mobility group box 1 proteins, and soluble hyaluronan (5, 39), all of which can serve as TLR4 ligands and activate NF-κB signaling (40). In addition, TLR4/NF-κB has been reported to regulate macrophage polarization (41). For example, Ye et al. reported that activation of the TLR4/NF-κB pathway increases polarization toward pro-inflammatory like macrophages (41, 42), and Gong et al. reported that inhibition of the TLR4/NF-κB signaling pathway inhibits polarization toward pro-inflammatory like macrophages (43). Thus, we concluded that differentiation of macrophages into pro-inflammatory like phenotypes after SCI is regulated via the DAMPs/TLR4/NF-κβ pathway. Furthermore, regarding the mechanism by which zinc inhibits the nuclear translocation of NF-κB, it is known that zinc directly inhibits IKK, which is located upstream of NF-κB and phosphorylates the NF-κB dimer to promote nuclear translocation of p65 (44). It has also been widely reported that direct inhibition of the IKK complex with zinc suppresses NF-κB activation and subsequent expression of inflammatory cytokines (12, 15, 45–47). In the present study, zinc deficiency increased the number of nuclear translocations of NF-κB, the number of pro-inflammatory like macrophages, and the subsequent expression of pro-inflammatory cytokines, and zinc supplementation improved these responses. Therefore, zinc would control the DAMPs/TLR4/NF-κβ pathway, which regulates the differentiation of macrophages into pro-inflammatory like phenotypes after SCI, via direct inhibition of IKK.

There are multiple views on the phenotype of macrophages and the issue is still controversial (48). Traditionally, they have been classified as pro-inflammatory M1 macrophages and anti-inflammatory M2 macrophages, but this classification has been pointed out as an oversimplification (49). It has also been noted that M1 and M2 macrophages are two polar forms of *in vitro* differentiated mononuclear phagocytes with different phenotypic patterns and functions, while *in vivo* there are various phenotypes in between, depending on the microenvironment and natural signals to which the macrophages are exposed (49). In addition, macrophages are reported to be highly plastic in response to microenvironmental stimuli and thus may therefore exhibit a variety of different immune phenotypes with overlapping properties (49, 50). Furthermore, macrophages have been reported not to be clearly divided into M1/M2 categories (51). Indeed, in this study macrophages also show increased anti-inflammatory markers as well as increased pro-inflammatory markers after SCI, suggesting that macrophages may exhibit different phenotypes with overlapping properties. Therefore, in this study we used the terms “pro-inflammatory like macrophage” and “anti-inflammatory like macrophage” to describe macrophage polarization rather than the traditional terms M1/M2 macrophages.

NF-κB is a transcription factor that normally plays a pro-inflammatory role, but is also known to suppress anti-inflammatory macrophage markers (52). In the present study, despite the activation of NF-κB by zinc deficiency, there was no difference in anti-inflammatory markers between the three groups of ZA, ZD, and ZDS (**Figure 1J**), which could be due to several factors. First, NF-κB activation occurs in the early phase of the inflammatory response, but the expression of anti-inflammatory markers usually increases in the latter phase of the inflammatory response, so it is possible that no difference in anti-inflammatory marker had yet appeared in this study, which was observed in the acute inflammatory phase on day 3 of SCI (53). Second, the regulation of the inflammatory response involves not only NF-κB but also other anti-inflammatory transcription factors and signaling pathways such as PPARγ and STAT3, which may have been affected by these factors (50). Although there were no differences in anti-inflammatory markers between the three groups ZA, ZD and ZDS, the anti-inflammatory markers in the three groups were increased compared to naïve, and when taken together with the increase in inflammatory markers, the results support the idea that there are different phenotypes of macrophages with overlapping properties (49).

Although the existence of zinc has long been known, little was known about how it functions in the body until recently (18, 23). This was due to the difficulty of conventional zinc measurement methods. Conventional measurement methods such as atomic absorption spectrometry or inductively coupled plasma optical emission spectroscopy (ICP-OES) require large amounts of samples and the cost of the equipment was very high (3, 54). However, the newly developed measurement kit can measure zinc concentration easily and inexpensively by applying absorbance measurement (54). In addition, the zinc indicator FluoZin3 allows the visualization of zinc presence and the evaluation of intracellular zinc concentration levels when applied in flow cytometry (3, 12). Due to the establishment of these measurement and visualization methods, zinc research has advanced dramatically in recent years, and zinc is now highlighted as a new therapeutic target in a wide variety of diseases (9, 23, 35).

Although the exacerbating factors that are amenable to treatment for SCI are not fully understood, we have previously reported that acute phase glycemic control improved functional outcome of SCI with attenuated microglial inflammatory response and subsequent demyelination (5). Since we have now demonstrated that low zinc is exacerbating factor and that zinc supplementation is effective in SCI, we expect to further improve functional outcome by regulating not only hyperglycemia but also the low zinc status.

To date, numerous studies have shown that zinc is relatively harmless compared to other heavy metals with similar properties (55). For example, Léonard et al. reported that zinc is not carcinogenic, teratogenic, mutagenic, or cytotoxic (56). In fact, zinc poisoning is reported to be very rare (9). This is because the estimated LD50 for humans, the amount that causes death in half of them, is 27 g zinc/day, which is considerably larger than the recommended dietary intake of zinc (11 mg/day for men and 8 mg/day for women) and the amount emitted (about 225-400 mg) (57, 58). Also, the LD50 of zinc is more than 10 times higher than that of cadmium and 50 times higher than that of mercury, which is quite a large amount, so lethal dose ingestion is highly unlikely (59). Moreover, in addition to acute poisoning, some have reported that long-term high-dose zinc supplementation interferes with copper intake, and that many of its toxic effects are actually due to copper deficiency rather than zinc itself (60). However, this zinc-induced copper deficiency has been reported to be totally reversible when zinc administration is stopped, and the time from zinc administration to the onset of copper deficiency has often been reported to be several months or years (61, 62). Considering that copper deficiency symptoms did not appear after 6 weeks of zinc administration in this study and that 6 weeks after SCI, when motor function mainly improves, is a sufficient time for zinc administration, this zinc administration method is reasonable because zinc administration can be stopped before symptoms appear. Thus, many studies have shown zinc to be a safe essential trace element. Furthermore, considering that zinc is inexpensive, the measurement of serum zinc concentration is simple, and administration methods such as oral and intravascular administration are well established, we believe that zinc is easy to be applied clinically in actual practice as a novel therapeutic agent for SCI.

## Conclusion

Zinc deficiency exacerbated motor functional outcome after SCI by promoting nuclear translocation of NF-κB, resulting in macrophage polarization to express increased pro-inflammatory cytokines. Zinc supplementation ameliorated these responses, thereby improving motor function, indicating that zinc supplementation could be a novel treatment after SCI.

## Data availability statement

The raw data supporting the conclusions of this article will be made available by the authors, without undue reservation.

## Ethics statement

Ethical approval was not required for the studies on humans in accordance with the local legislation and institutional requirements because only commercially available established cell lines were used. The animal study was approved by the Committee of Ethics on Animal Experimentation in the Faculty on Medicine, Kyushu University (A-29-243-0). The study was conducted in accordance with the local legislation and institutional requirements.

## Author contributions

KKij: Conceptualization, Data curation, Formal Analysis, Funding acquisition, Investigation, Methodology, Project administration, Resources, Visualization, Writing – original draft, Software, Writing – review & editing. GO: Formal Analysis, Writing – original draft, Data curation, Investigation, Visualization. KKob: Conceptualization, Funding acquisition, Project administration, Resources, Supervision, Validation, Writing – review & editing, Software, Methodology. HSai: Conceptualization, Funding acquisition, Project administration, Resources, Supervision, Validation, Writing – review & editing, Software, Methodology. MH: Conceptualization, Formal Analysis, Methodology, Supervision, Writing – original draft, Data curation, Investigation. SY: Formal Analysis, Writing – original draft, Data curation. KY: Formal Analysis, Writing – original draft, Methodology. TS: Data curation, Formal Analysis, Writing – original draft. TT: Data curation, Formal Analysis, Writing – original draft. HI: Data curation, Formal Analysis, Writing – original draft. YH: Formal Analysis, Writing – original draft, Data curation. KKit: Formal Analysis, Writing – original draft, Data curation. TU: Conceptualization, Formal Analysis, Supervision, Validation, Writing – review & editing, Methodology. DK: Conceptualization, Data curation, Formal Analysis, Methodology, Project administration, Supervision, Writing – original draft. VE: Conceptualization, Supervision, Validation, Writing – review & editing. CL: Supervision, Validation, Writing – review & editing. HSak: Data curation, Methodology, Resources, Writing – original draft. TM: Data curation, Supervision, Writing – original draft, Resources. KKaw: Data curation, Supervision, Writing – original draft. YM: Supervision, Validation, Writing – review & editing. SO: Conceptualization, Data curation, Funding acquisition, Methodology, Project administration, Resources, Supervision, Validation, Writing – review & editing, Formal Analysis, Investigation. YN: Data curation, Project administration, Resources, Supervision, Validation, Writing – review & editing, Software.
